# A single nucleotide polymorphism causes enhanced radical oxygen species production by human aldehyde oxidase

**DOI:** 10.1371/journal.pone.0182061

**Published:** 2017-07-27

**Authors:** Alessandro Foti, Frank Dorendorf, Silke Leimkühler

**Affiliations:** Department of Molecular Enzymology, Institute of Biochemistry and Biology, University of Potsdam, Potsdam, Germany; Istituto di Genetica Molecolare, ITALY

## Abstract

Aldehyde oxidases (AOXs) are molybdo-flavoenzymes characterized by broad substrate specificity, oxidizing aromatic/aliphatic aldehydes into the corresponding carboxylic acids and hydroxylating various heteroaromatic rings. The enzymes use oxygen as the terminal electron acceptor and produce reduced oxygen species during turnover. The physiological function of mammalian AOX isoenzymes is still unclear, however, human AOX (hAOX1) is an emerging enzyme in phase-I drug metabolism. Indeed, the number of xenobiotics acting as hAOX1 substrates is increasing. Further, numerous single-nucleotide polymorphisms (SNPs) have been identified within the *hAOX1* gene. SNPs are a major source of inter-individual variability in the human population, and SNP-based amino acid exchanges in hAOX1 reportedly modulate the catalytic function of the enzyme in either a positive or negative fashion. In this report we selected ten novel SNPs resulting in amino acid exchanges in proximity to the FAD site of hAOX1 and characterized the purified enzymes after heterologous expression in *Escherichia coli*. The hAOX1 variants were characterized carefully by quantitative differences in their ability to produce superoxide radical. ROS represent prominent key molecules in physiological and pathological conditions in the cell. Our data reveal significant alterations in superoxide anion production among the variants. In particular the SNP-based amino acid exchange L438V in proximity to the isoalloxanzine ring of the FAD cofactor resulted in increased rate of superoxide radical production of 75%. Considering the high toxicity of the superoxide in the cell, the hAOX1-L438V SNP variant is an eventual candidate for critical or pathological roles of this natural variant within the human population.

## Introduction

Reactive oxygen species contribute to numerous physiological and pathological phenomena in all living organisms having an aerobic life and are generated by various systems in eukaryotic cells. Endogenous ROS are produced during mitochondrial oxidative metabolism due to the respiratory redox enzymes, as well as in cellular response to xenobiotics, cytokines and pathogens infection [1]. An enzyme that has been implicated in producing significant amount of ROS in eukaryotic cells is xanthine oxidase (XO).

Notably, a highly XO-related molybdoenzyme present in most living organisms is aldehyde oxidase (AOX, EC 1.2.3.1), having an amino acid sequence identity of 49.8% to XO and existing only as oxidase form. Both XO and AOX were suggested to be derived from a common ancestor by gene duplication events [2]. In their catalytically active forms AOX and XO exist as a homodimer with a molecular mass of 300 kDa. Each monomer consists of three domains: an N-terminal domain (20 kDa) containing two spectroscopically distinct [2Fe2S] centers, a 40 kDa intermediate domain containing a flavin adenine dinucleotide (FAD) binding site [3], and a C-terminal domain (85 kDa) containing the substrate binding pocket in addition to the molybdenum cofactor (Moco) binding site. Despite their remarkable similarities in cofactor composition, amino acid sequence, molecular mass, 3D structures, and common genetic origin, XO and AOX have different substrate and inhibitor specificities. While XO mainly is involved in the catabolism of purines producing uric acid from hypoxanthine and xanthine, AOX catalyzes the oxidative hydroxylation of a wider variety of substrates including a number of aliphatic and aromatic aldehydes, and nitrogen containing heterocyclic compounds. In contrast to XO, the biochemical and physiological functions of mammalian AOXs are still unclear [4]. Nevertheless, human AOX1 is an enzyme of emerging relevance for phase I drug metabolism [2,5]. Indeed, the number of xenobiotics acting as human AOX1 substrates is increasing. In particular, the current trend in drug development focusing on the design and synthesis of molecules that are not recognized and inactivated by cytochrome P-450 dependent monoxygenases has resulted in an enrichment of molecules which can be oxidized by AOXs [2]. In humans, the main source of AOX is the liver [6] but the enzyme is also present in lungs, digestive tract, urinary and sexual organs, spinal cord and central nervous system [7,8,9]. AOX shows an important role in the metabolism of drugs and xenobiotics in the human hepatocytes [7]. Overall, AOX exclusively exists as the oxidase form using molecular oxygen as electron acceptor and cannot transfer electrons to NAD^+^ at its FAD site.

The recently solved crystal structure of human AOX1 (hAOX1) enabled direct comparison of the FAD sites of both enzymes [10]. In hAOX1, the FAD cofactor is located in the middle domain of the enzyme, with the isoalloxazine ring stacked between two leucine residues (344 and 438) [10]. Notably, hAOX1 and the dehydrogenase form of bovine XDH (bXDH) showed a similar conformation of a variable loop at the FAD site (named variable loop I) ranging from amino acids 430–440, which is implicated in the coordination of the FAD cofactor and in the XO/XDH interconversion. In the XO/XDH interconversion, the XDH form which preferentially interacts with NAD^+^ as terminal electron acceptor is converted to the XO form, which exclusively interacts with O_2_ as electron acceptor. In contrast, hAOX1 showed a different conformation of a second loop at the FAD site ranging from amino acids 1230–1235 (named variable loop II). At the entrance of the FAD pocket, loop II T_1230_RGPDQ_1235_ is flipped almost 180° in comparison to the corresponding loop in bXO and bXDH. This loop takes a conformation so far only found in hAOX1, and sits approximately at the same position as the nicotinamide ring of the bXDH-NADH complex structure blocking the access to the isoalloxazine ring [11]. AOX has been suggested to be a source of ROS and further, a role of the generation of nitric oxide (NO) from nitrite reduction during ischemia has also been suggested [12]. Thus, in addition to the metabolism of drugs, AOX could serve as an important biological source of ROS, NO, and reactive nitrogen species (RNS) and might play a crucial role in ROS or RNS mediated signaling and tissue injury [12]. While previous investigations on the reactivity of AOX were mainly based on studies using rat or rabbit enzymes, investigations on the ROS production by the human enzyme have not been performed so far. Differences in enzymatic activities using human AOX1 and the AOX enzymes from rodents or other species are expected, since these species contain a different set of AOX isoenzymes.

However, among the human population also an intra-specific diversity is present effecting the expression, the tissue distribution and the enzymatic activity of hAOX1. In humans, the above-mentioned differences between individuals were considerably attributed to single nucleotide polymorphisms (SNPs) in the *AOX1* gene. An increasing number of SNP variations in the *AOX1* genomic sequence are listed in the NCBI SNP gene data bank (http://www.ncbi.nlm.nih.gov/snp) and several of these polymorphisms were identified to be located in the area surrounding the FAD site.

Here, we selected ten novel SNPs resulting in amino acid exchanges in proximity to the FAD site of hAOX1 and characterized the purified enzymes after heterologous expression in *Escherichia coli*. We studied the kinetic and spectroscopic properties of the hAOX1 SNP-based variants and determined the levels of ROS generated by each enzyme. Our data reveal significant alterations in superoxide anion production. The data point out an important role of selected amino acid residues in oxygen radical formation at the FAD active site of the hAOX1, uncovering physiological consequences in individuals carrying SNPs at this site.

## Results

### Purification of hAOX1 variants containing SNP-based amino acid exchanges

**Fig 1:**
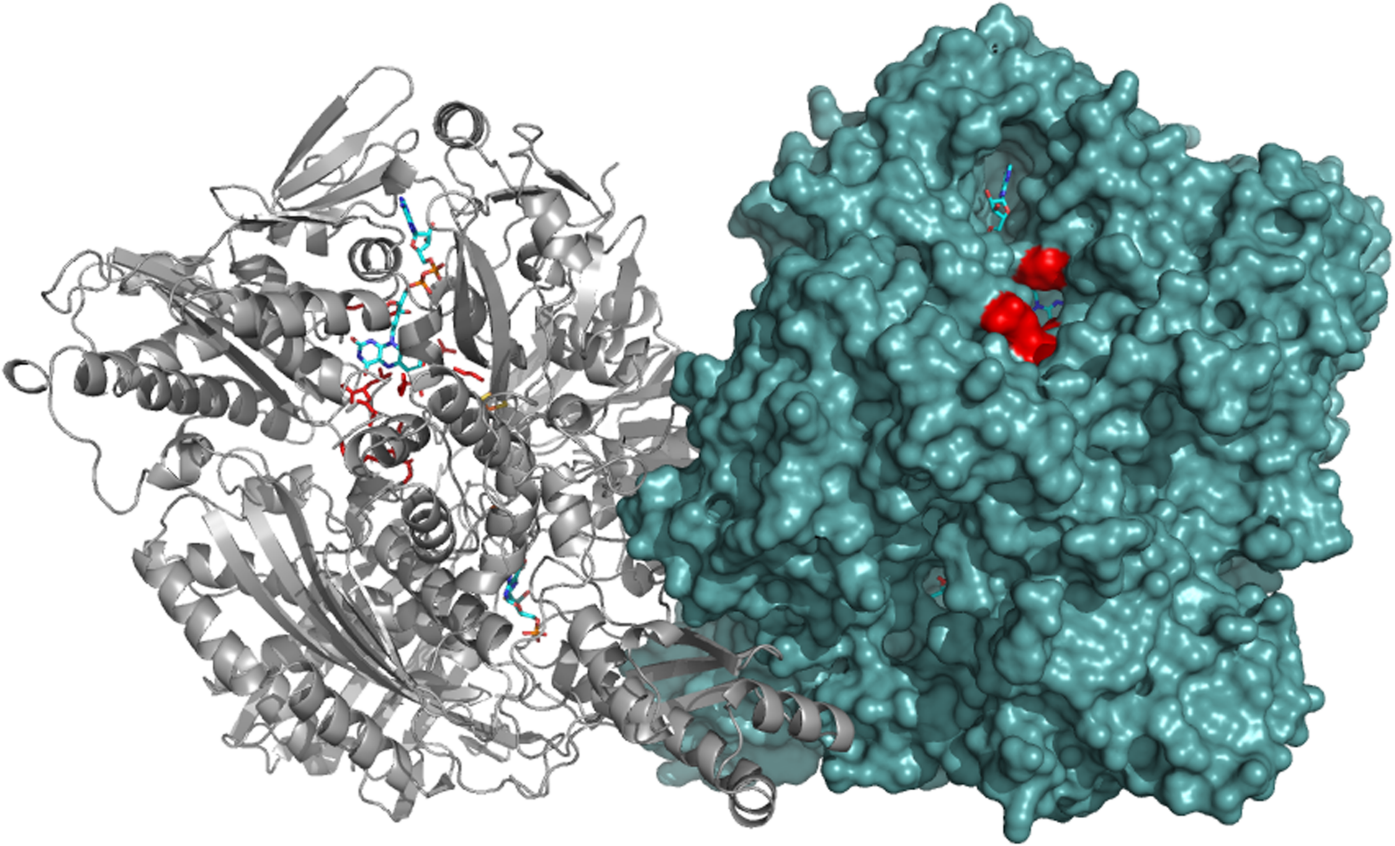
Overall structure of the hAOX1 dimer and location of the SNP-based amino acid exchanges. Monomer A is shown as surface representation (right) and the monomer B as cartoon (left) showing the Moco, 2x[2Fe2S] centers and the FAD cofactor. In close proximity to the FAD cofactor the location of the SNP-based amino acid exchanges characterized in this study are shown as red sticks. The figure was generated using the PDB code 1UHW published by Coelho et al., 2015.

**Fig 2:**
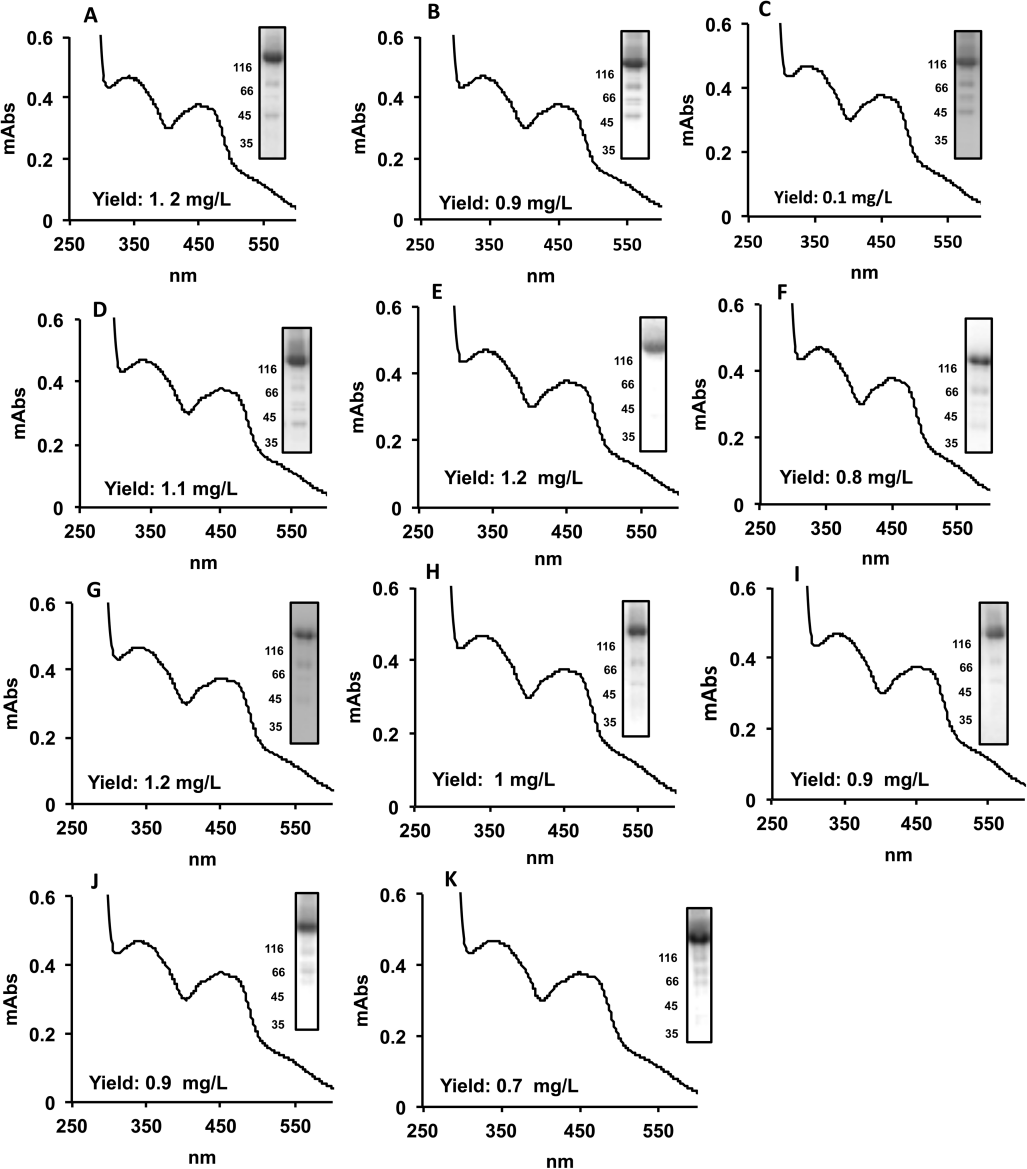
Characterization of the hAOX1 SNP-based variants. The main graphs illustrate the UV-Vis spectra of the indicated proteins isolated from *E*. *coli*. The UV-Vis spectra were recorded using purified air-oxidized hAOX1 proteins in 50 mM Tris, pH 8.0. The yield of protein after purification is indicated. (A) hAOX1 WT; (B) hAOX1 G46E; (C) hAOX1 G50D; (D) hAOX1 G346R; (E) hAOX1 H363Q; (F) hAOX1 R433P; (G) hAOX1 A437V; (H) hAOX1 L438V; (I) hAOX1 A439E; (J) hAOX1 hAOX1 R1231H; (K) hAOX1 K1237N. The 10% SDS-polyacrylamide gels of the indicated proteins after staining with Coomassie blue are shown in the rightmost inset of each panel. The majority of the lower bands shown in the SDS-PAGE gels were identified by mass spectrometry as degradation products of hAOX1 as reported before (Hartmann et al., 2012).

The human AOX1 gene consists of 35 coding exons resulting in a protein of 1338 amino acids [7]. In the SNP database an increasing number of non-synonymous coding variations are described (https://www.ncbi.nlm.nih.gov/ protein/XP_011509364.1). By aid of the available crystal structure for hAOX1, we selected SNPs resulting in amino acid exchanges around the FAD active site and in proximity to FeSII for more detailed investigations (Fig 1). The reported hAOX1 SNP-based variants can be grouped in three categories: *i*) amino acid exchanges located on the FAD variable loop 1 and 2 (R1231H, A439E, R433P, L438V, A437V, K1237N); *ii*) amino acid exchanges in close proximity of the FAD cofactor (G346R, H363Q); and *iii*) amino acid exchanges in between the FeSII and the FAD cofactor (G46E, G50D) (Fig 1). These corresponding base pair exchanges were introduced into the *AOX1* gene and together with the wild-type enzyme were recombinantly expressed and purified according to the previously published procedure [13]. The general protein yield was about 1 mg of protein per liter of *E*. *coli* culture for each variant. After size exclusion chromatography step all hAOX1 variants eluted mainly as a dimer with a molecular mass of 300 kDa (data not shown) and the Coomassie-stained SDS polyacrylamide gels generally showed that all variants were obtained with high and comparable purities (Fig 2). The only exception is the variant hAOX1-G50D, which was purified with a 10-fold lower yield as compared to the other variants.

### Quantification of the cofactor loading levels of the purified hAOX1 variants

To compare the overall characteristics of the generated hAOX1 variants, UV-Vis spectra were recorded (Fig 2). The UV-Vis spectra of hAOX1 and the variants in their oxidized forms displayed the typical features of molybdo-flavoenzymes with a prominent absorption maximum at 450 nm and a shoulder at 550 nm, representing the presence of FAD and the two [2Fe2S] clusters. The saturation levels of the enzymes with molybdenum, iron and FAD were additionally determined (Table 1) and show that all variants have a comparable saturation level with molybdenum around 56%. The enzymes were additionally compared by their saturation with Mo-MPT cofactors after conversion to its fluorescent derivative (FormA), to exclude the unspecific incorporation with molybdenum, and the results showed comparable levels of the cofactor in consistency with the molybdenum levels (data not shown). The saturation levels with iron were around 65% for all the enzymes and reflected the saturation with 2x[2Fe2S] clusters. Additionally, the loading of the enzymes with FAD was quantified. The results in Table 1 show that all hAOX1 variants were saturated with FAD around 85% similar to the hAOX1 wild-type, revealing that the introduced amino acid exchanges did not influence the overall cofactor saturation levels of the enzymes.

**Table 1:** Steady state kinetic parameters of hAOX1 wild-type and variants and data for saturation with molybdenum, iron and FAD.

Enzyme	Mo content (%)	Fe content (%)	FAD saturation (%)	^a^k_cat_ (min^-1^)	K_M_ (μM)
hAOX1 wild-type	62 ± 9	69 ± 8	93 ± 4	273 ± 36	9 ± 4
G46E	55 ± 6	66 ± 5	85 ± 7	534 ± 35	14 ± 5
G50D	63 ± 7	58 ± 13	85 ± 10	58 ± 15	12 ± 4
G346R	49 ± 7	62 ± 6	89 ± 5	61 ±17	6 ± 1
H363Q	56 ± 11	71 ± 3	90 ± 3	271 ± 23	13 ± 4
R433P	65 ± 12	67 ± 5	79 ± 6	68 ± 4	8 ± 1
A437V	61 ± 5	72 ± 4	83 ± 6	281 ± 23	11 ± 3
L438V	49 ± 10	65 ± 5	95 ± 2	343 ± 59	10 ± 3
A439E	51 ± 8	71 ± 6	86 ± 5	49 ± 3	10 ± 2
R1231H	57 ± 9	64 ± 5	76 ± 12	132 ± 46	9 ± 2
K1237N	48 ± 6	65 ± 7	79 ± 9	76 ± 6	6 ± 2

^a^Phenanthridine was used as substrate and molecular oxygen as electron acceptor. k_cat_ values were corrected to a molybdenum saturation of 100%

### Steady state kinetics of hAOX1 variants

To compare the kinetic parameters of hAOX1 wild-type and the variants steady-state kinetics with phenanthridine as substrate and oxygen as electron acceptor were performed. The kinetics constants were determined by quantification of the product phenanthridinone following the absorbance increase at 321 nm. The K_M_ and k_cat_ values calculated for each hAOX1 variant are shown in Table 1. To obtain better comparability of the data among the variants, the kinetic constants were corrected and normalized for a 100% molybdenum saturation level of the purified enzymes (Table 1). While the K_M_ values of the variants were comparable to the one of the wild-type enzyme and ranged around 6–14 μM, the k_cat_ values showed some differences. In the case of variants G50D, G346R, R433P, A439E, R1231H and K1237N, k_cat_ was significantly reduced with values around 20–50% of the value determined for the wild type enzyme. In contrast, the amino acid exchanges G46E and L438V resulted in a 2-fold and 1.2-fold increase in k_cat_, respectively, while the k_cat_ of variants H363Q and A437V was not changed in comparison to the wild-type enzyme.

### Inhibition of the electron transfer at the FAD site with diphenyleniodonium (DPI)

**Fig 3:**
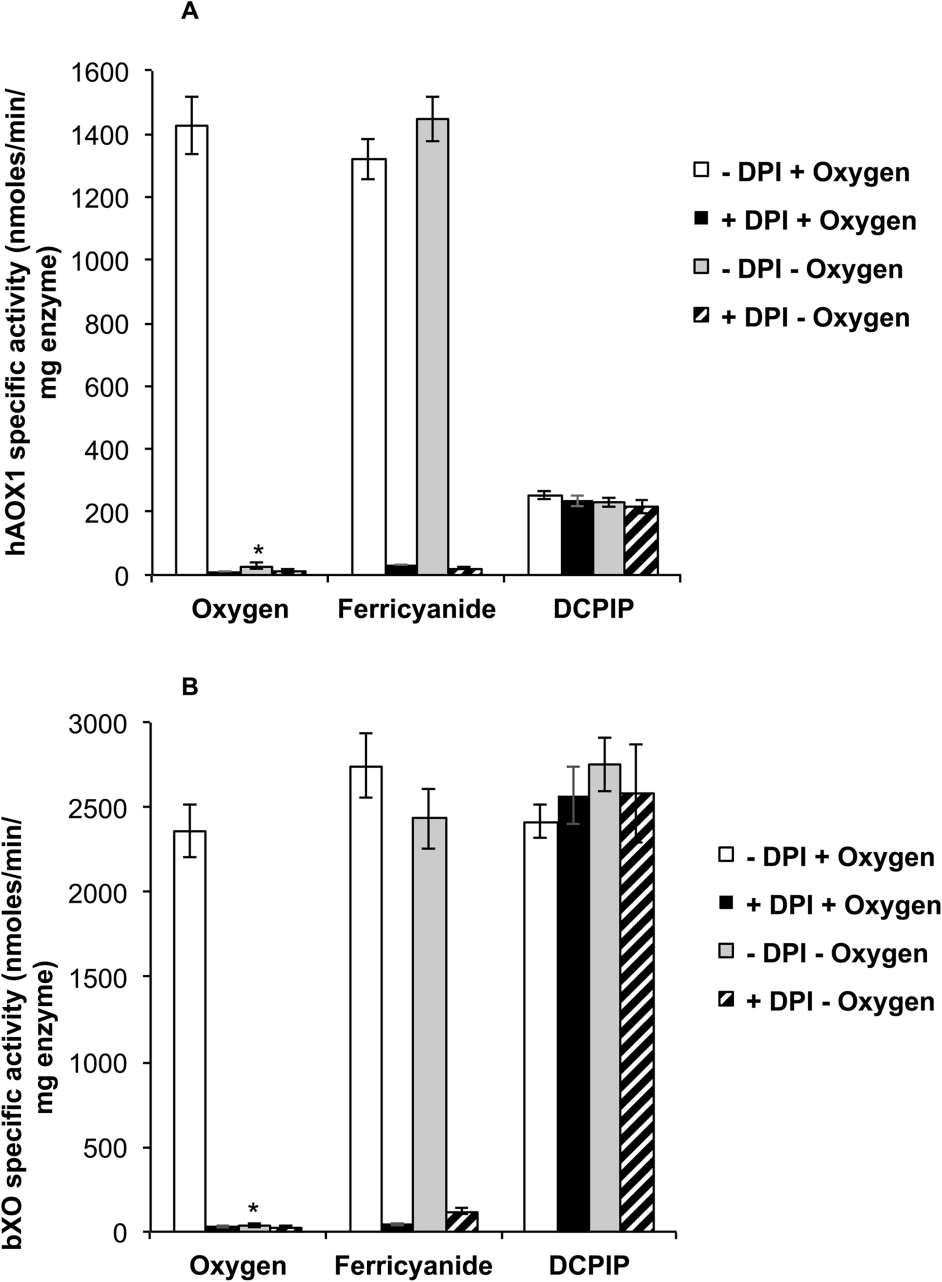
The effect of diphenyleiodonium on hAOX1 activity. Specific activities of (A) hAOX wild-type with 80 μM phenanthrine as substrate and (B) of bXO with 50 μM xanthine as substrate. Assays were performed using molecular oxygen (air-saturated buffer) or 500 μM ferricyanide and 100 μM 2,6-dichlorophenolindophenol (DCPIP) as electron acceptors either under aerobic or under anaerobic conditions. The effect of 5 μM diphenyleneiodonium (DPI) on enzyme activity was assayed under these conditions.

We further compared the functional reactivities of the electron acceptors oxygen, ferricyanide and DCPIP, with hAOX1 wild-type and the diphenyleniodonium (DPI) inhibited enzyme. DPI is a well-known inhibitor of flavoenzymes, which covalently binds to the FAD/FMN sites and consequently blocks the electron transfer activity of the flavin cofactors [14]. The results in Fig 3 show the data obtained for hAOX1 wild-type in comparison to the ones obtained for the bXO enzyme. For both enzymes the obtained specific activities show that the xanthine/phenanthridine:O_2_ and xanthine/phenanthridine:ferricyanide reactivities were inhibited by DPI, while the xanthine/phenanthridine:DCPIP activity was not influenced by the presence of DPI in the assay. This shows that both O_2_ and ferricyanide are accepting the electrons at the FAD site of hAOX1 and bXO, while DCPIP directly reacts at the Moco active site, as suggested previously [15]. In contrast to bXO, hAOX1 showed a significantly reduced specific activity with DCPIP as electron acceptor, confirming that DCPIP is an inhibitor of hAOX1, but not of bXO [13]. The detected uncompetitive inhibition mode of hAOX1 with DCPIP likely shows that DCPIP reacts with the reduced form of Moco.

**Fig 4:**
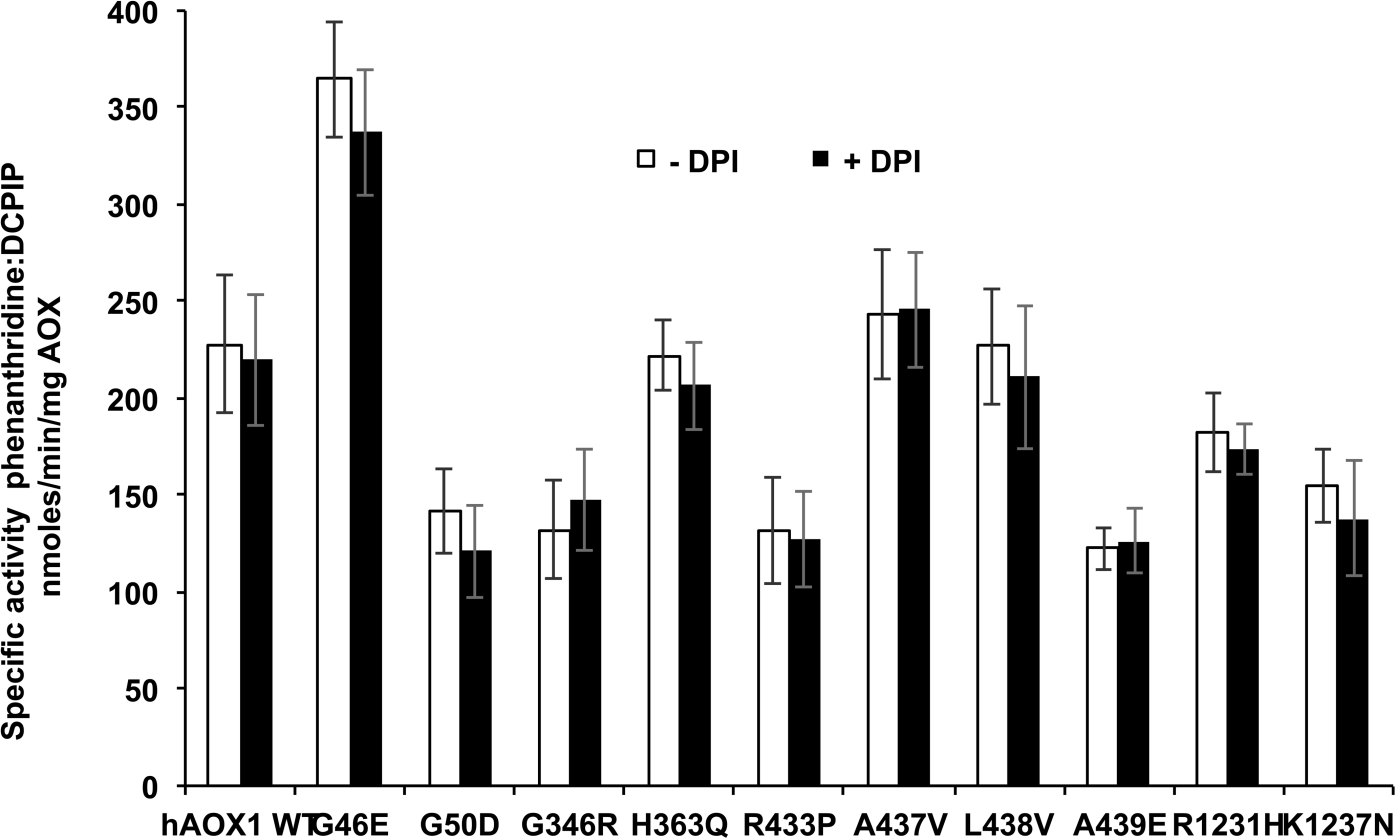
The effect of diphenyleiodonium on the activity of hAOX1 variants. Specific activities of hAOX1 wild-type and SNP-based variants with 80 μM phenanthridine as substrate and 100 μM 2,6-dichlorophenolindophenol (DCPIP) as electron acceptor in absence or presence of 5 μM diphenyleneiodonium (DPI).

To analyze the influences of the amino acid exchanges on the enzyme activity, we determined the phenanthridine:DCPIP reactivities of the variants in the presence and absence of DPI. The phenanthridine:DCPIP reaction (especially of the DPI-FAD inhibited enzymes) directly occurs at the Moco site and does not involve intramolecular electron transfer and therefore a direct comparison with the data of the phenanthridine:O_2_ activities was carried out (Table 1). The data give insights whether the impact of the mutation effects the intramolecular electron transfer or the electron transfer from FAD to oxygen. The results in Fig 4 show that the specific activities of all hAOX1 variants were not influenced by the presence of DPI. In similarity with the turnover numbers determined for the oxygen reactivity, the specific activities with DCPIP show some differences among the variants (Fig 4). In the case of variants G50D, G346R, R433P, A439E, R1231H and K1237N specific activities were reduced with values around 50–75% of the value determined for the wild type enzyme. In contrast, the amino acid exchange G46E resulted in a 1.5-fold increase in specific activity, while the values of variants H363Q, A437V and L438V were comparable to the ones of the wild-type. In total, these results show that the differences in activities detected for the variants are not based on an altered electron transfer from FAD to O_2_ but rather are based on differences at the Moco site, which might be due to different sulfuration levels of the enzyme variants.

**Fig 5:**
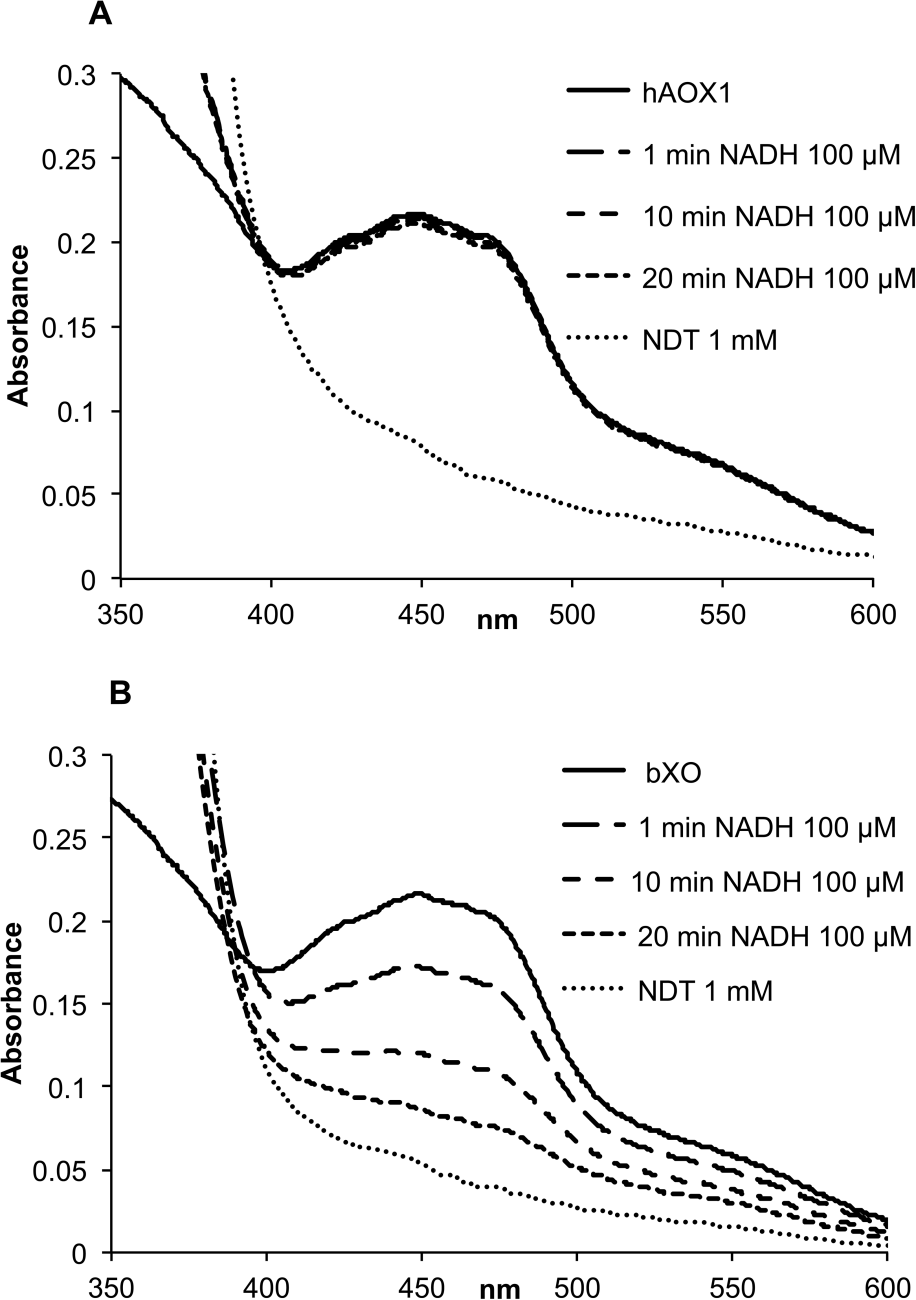
UV-Vis spectra of hAOX1 in comparison to bXO reduced with NADH under anaerobic conditions. The figure illustrates UV-Vis spectra of 10 μM hAOX1 and 10μM bXO in the oxidized state (solid line) and reduced enzyme with 0.1 mM NADH after 1 min, 10 min and 20 min (dashed lines) and of the enzyme reduced with 1 mM sodium dithionite (NDT) (dotted line). The spectra were recorded in 50 mM Tris, pH 8.0.

Furthermore, hAOX1 was assayed for NADH oxidation activity in comparison to bXO. As shown in Fig 5, hAOX1 incubated in presence of 100 μM NADH did not show reduction of the FAD over an incubation time of 20 min, while bXO was readily reduced. This result shows the inability of hAOX1 to oxidize NADH.

### Superoxide formation by hAOX1 and SNP based variants

**Fig 6:**
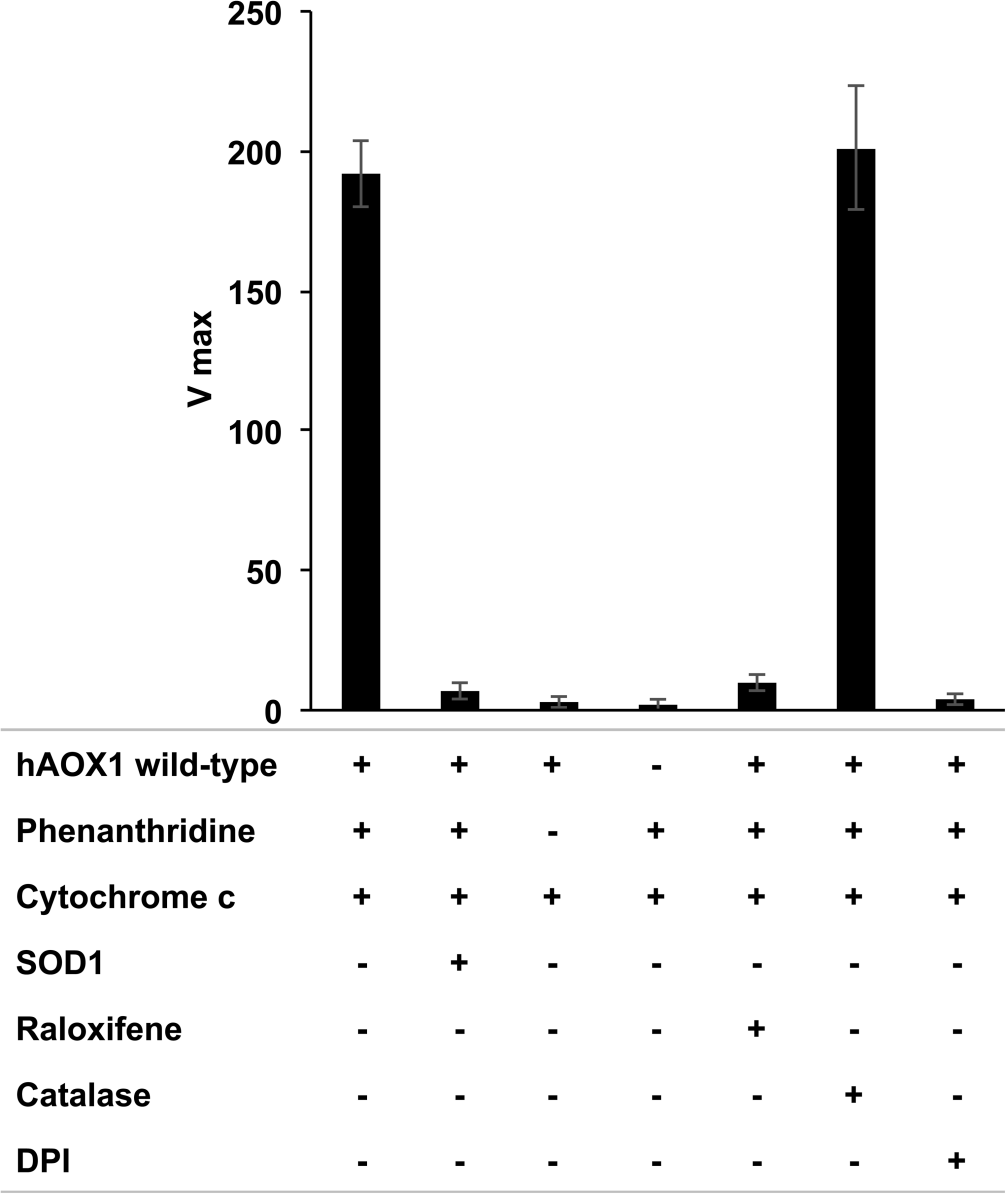
Superoxide production by hAOX1 wild-type. Superoxide production of enzymes were calculated by following the reduction of 100 μM cytochrome c at 550 nm in the presence of 50 μM phenanthridine in 50 mM Tris, pH 8.0. 20 μM superoxide dismutase 1 (SOD1), 10 μM raloxifene, 25 μM catalase and 5 μM diphenyleneiodonium (DPI) were included in the assays as indicated.

AOXs have been reported to produce H_2_O_2_ as the final reaction product, however, studies on the amount of superoxide production of the purified human enzyme have not been performed so far. The production of O_2_^•−^ can be conveniently monitored by including cytochrome-c in the reaction mixture. In fact, cytochrome-c reduction by O_2_^•−^ is considerably faster than spontaneous O_2_^•−^ dismutation. The amount of O_2_^•−^ produced in the reaction mixture can be estimated by comparison to a blank reaction run under the same conditions in the presence of superoxide dismutase. The reduction of cytochrome c was almost completely quenched by superoxide dismutase 1 (Fig 6). Since hAOX1 also catalyzes the two-electron reduction of molecular oxygen to H_2_O_2_, which in turn can re-oxidize Fe^2+^cytochrome c to Fe^3+^cytochrome c, the reaction was performed in the presence of catalase (100 U/mL) that completely decomposes H_2_O_2_ (Fig 6). To confirm further that the observed cytochrome c reduction is based on the activity of hAOX1, the reaction was carried out in the presence of the inhibitor raloxifene, which readily inhibited the reaction. As further control, the FAD site was inhibited with DPI, which completely abolished cytochrome c reduction. In addition to the reduction of cytochrome c, another assay was applied for superoxide detection, which is based on the reduction of nitroblue tetrazolium (NBT) to formazan [16]. By this assay comparative results with the cytochrome c reduction assay were obtained (data not shown).

To obtain the percentage of O_2_^•−^ production for hAOX1 wild-type and the variants, the reduction of cytochrome c (U/μmol enzyme) was related to the overall rate of phenanthridinone production (U/μmol enzyme) (Table 2). The hAOX1 wild-type enzyme is characterized by a percentage of superoxide production of 11%±3. While the variants G46E, G50D and R433P showed similar values to the wild-type enzyme, the rate of superoxide production was slightly increased in the variants G346R, A439E, R1231H and K1237N with ratios around 15–18% and an even higher increased rate in variant A437V with 25%. Interestingly, a significantly increased rate of superoxide production was obtained for the variant L438V with a rate of 72%±10. The leucine residue in position 438 is located within the flexible loop I (Q_430_AQRQENALAI_440_) and in proximity of 3.42 Å to the N5 of the isoalloxazine ring (being stacked between residues L344 and L438). In addition to phenanthridine, the rate of superoxide production was also followed with phthalazine and 4-dimethylaminocinnamaldehyde as substrates, resulting in comparable numbers of superoxide produced (data not shown).

**Table 2:** Superoxide production of hAOX1 wild-type and variants.

Enzyme	Specific activity phenanthridine:O_2_ (nmoles/min/mg of enzyme)	Specific activity phenanthridine:cyt c (nmoles/min/mg of enzyme)	O_2_^.-^ production ratio (%)
hAOX1 wild-type	1148 ± 151	126 ± 16	11 ± 3
G46E	2131 ± 177	256 ± 31	12 ± 3
G50D	272 ± 57	28 ± 7	10 ± 4
G346R	302 ± 41	45 ± 9	15 ± 3
H363Q	1291 ±73	168 ± 36	13 ± 11
R433P	266 ± 31	32 ± 6	12 ± 2
A437V	1192 ± 95	298 ± 39	25 ± 3
L438V	1423 ± 193	1025 ± 141	72 ± 10
A439E	209 ± 45	33 ± 5	16 ± 4
R1231H	689 ± 104	124 ± 35	18 ± 7

## Discussion

Here we report on the characterization of SNP based hAOX1 variants surrounding the FAD site and show their effect on the electron transfer pathway and oxygen radical formation. The major aim of the study was to determine the rate of superoxide production by hAOX1 and to analyze alterations based on amino acid exchanges of natural variants present within the human population.

ROS represent prominent key molecules in physiological and pathological conditions in the cell. Under controlled conditions, ROS are essential species involved in homeostatic cellular processes as cell cycle, redox signaling and defense against pathogens [1,17,18]. In contrast, when ROS achieve higher and unbalanced concentrations, they are a dangerous source of damage to several molecules within the cell. ROS can cause damage to the DNA, leading to an oncogenic effect, damage to lipids with consequent peroxidation and damage to residues or cofactors of proteins, e.g. iron-sulfur clusters [17]. So far mainly XOR has been implicated to generate significant amounts of O_2_^•−^ during the course of their catalytic activity against recognized substrates [19]. A report by Kundu et al. in 2012 suggested, however, that hAOX1 represents a significant source of ROS in the cytosol and plays a critical role in in ROS-mediated tissue injury under specific conditions [12]. This suggestion was based on the levels of enzymatic activity of XOR and AOX in human liver, where AOX1 has been calculated to have 24-fold higher enzyme activity than XOR [20]. However, the reports by Kundu et al. in 2007 [21] and 2012 [12] investigated the rat liver AOX enzymes, which represented a mixture of all four AOX isoenzymes since the isoenzymes were not separated during purification. All further existing studies investigating the amount of ROS production by AOX enzymes in the literature were also performed on either the rat, rabbit or mouse enzymes, species containing various isoenzymes with different activities which were not investigated separately. Studies on the rate of superoxide production by the human AOX1 enzyme have not been performed so far. In our study, the wild-type hAOX1 enzyme was shown to produce superoxide radical with a rate of around 10% as compared to the overall reaction. This value, however, is lower than the reported rates of 16–20% for bXO and significantly lower than the rates of 32% for the rat AOXs [12]. Thus, hAOX1 as major superoxide producing source should be analyzed carefully, since the k_cat_ = 273 min^-1^ with phenanthridine of the enzyme is significantly lower than the k_cat_ = 1100 min^-1^ of human XO with xanthine [22]. However, hAOX1 exists always in the oxidase form and does not need a conversion for oxygen reactivity and further exists in 24-fold higher amount than XOR in the cell, thus, being a factor to be considered for ROS generation in the cell.

**Fig 7:**
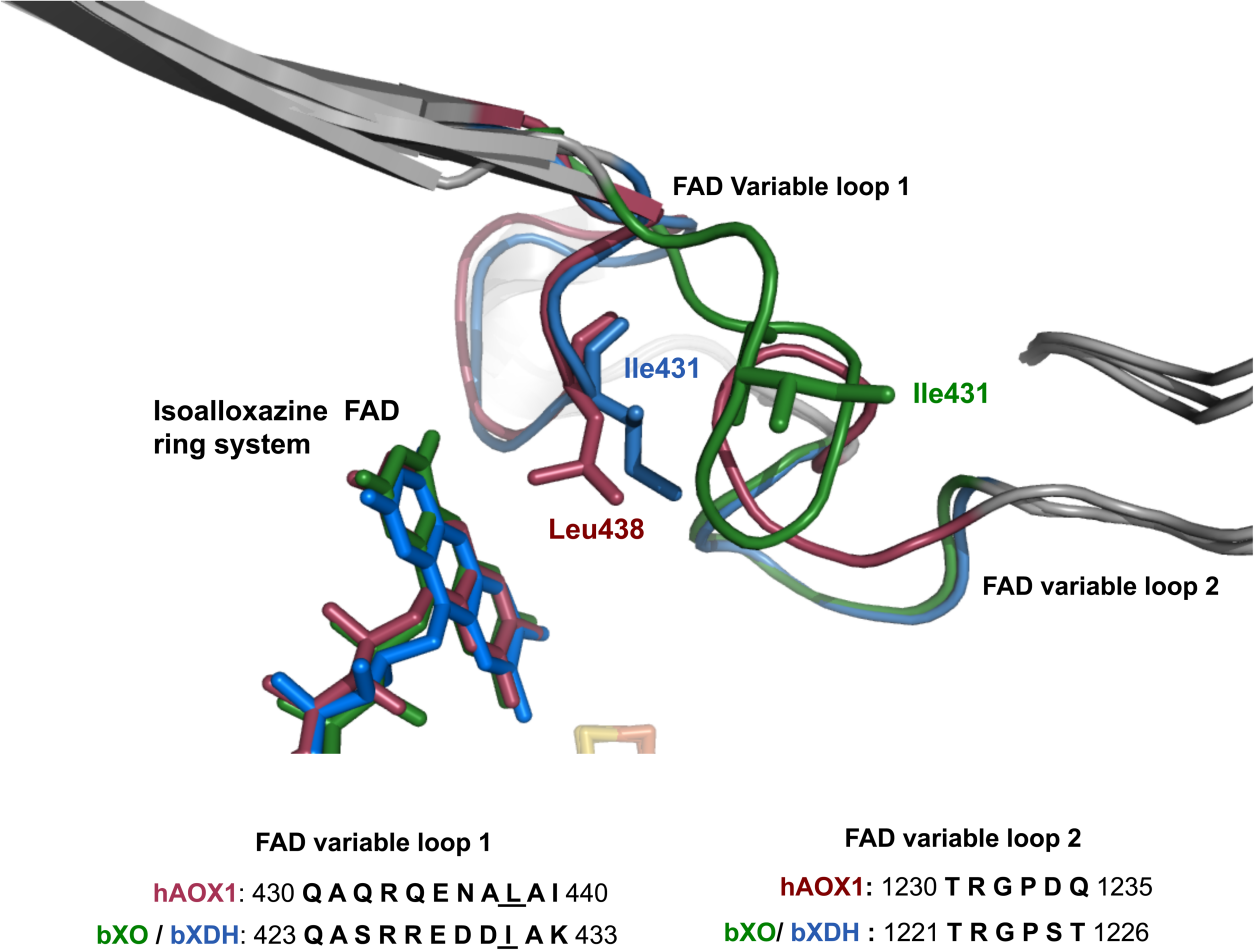
The FAD site of hAOX1. Close-up and superposition of the crystal structures of the FAD binding sites of hAOX1 (red, PDB ID: 4UHW), bXO (green, PDB ID: 1FIQ) and bXDH (blue, PDB ID: 1FO4). Shown is the location of the residues Leu438 of hAOX1 (dark red) and Ile431 of bXO/bXDH (green/blue). The FAD variable loops 1 and 2 are located in close proximity to the isoalloxazine ring of the FAD cofactors. The amino acid sequence alignment of the two loops is shown.

In the human population, a major source of inter-individual variability are nonsynonymous SNPs. So far, some SNPs were characterized on the protein level that were translated into AOX1 protein variants with increased catalytic activity, while other SNPs resulted in an inactive protein or AOX1 enzymatic variants with reduced catalytic activity [23]. In this study, we combined the study of hAOX1 SNPs variants and their effect on superoxide generation. A significant difference was identified in the rate of superoxide formation in one of the SNP-based variants in hAOX1. Among the ten SNPs located around the FAD site, the amino acid exchange L438V proved to be the most interesting one, since this variant produced a rate of around 75% of superoxide radical. The unbalanced superoxide production by the L438V amino acid exchange was unexpected considering the high similarity between the leucine and valine residues, both with a hydrophobic side chain but with valine having a shorter side chain (Fig 7). Considering the high toxicity of the superoxide anion in the cell, the hAOX1-L438V SNP variant it is an eventual candidate for critical or pathological roles of this natural variant within the human population. Alterations can be caused by high and uncontrolled oxygen radical production, particularly under pharmacological treatments where AOX is especially active and catalyzing the reduction of molecular oxygen in human liver hepatocytes. All other SNP based amino acid exchanges in the FAD loop I or loop II did not change the rate of superoxide production significantly, even when the amino acid exchange was located directly in vicinity to the amino acid L438. The basis of the significantly increased rate in superoxide production of the L438V variant, however, needs to be investigated in more detail.

By comparison, an amino acid substitution in rat XO has been reported which resulted also in an increased rate of superoxide production. A variant of rat XOR containing the amino acid exchanges W335A/F336L resulted in a form of the enzyme that is stably locked in the oxidase form. This enzyme variant was reported to produce O_2_^•−^ and H_2_O_2_ in a ratio of 6:1 [24]. The high rate of superoxide formation and the inability of the double variant to be converted to the dehydrogenase form has been explained by significant changes at the FAD site. Consequently, in the double variant a positive shift of the redox potential of the FAD cofactor was determined, with the result of the increased O_2_ reactivity of the enzyme. In native XOR, the reduction potential of the FeSI cluster and FAD are similar. Based on the thermodynamic equilibrium of the electrons introduced into the wild-type enzyme, the FAD cofactor was shown exists in both the FADH_2_ and FADH• states in XO, which was leading to both H_2_O_2_ and O_2_^•−^ production. Due to the increase in the FAD midpoint potential in the W335A/F336L variant, the electrons were explained to be more easily transferred from FeSI to the FAD in a one-electron transfer step, resulting in the formation of more FADH• and thus a higher O_2_^•−^ production rate. In AOX, however, stabilization of the FADH• has not been observed so far, thus, other factors than the stabilization of the FADH• contribute to the increased rate of O_2_^•−^ production.

For the *Arabidopsis thaliana* aldehyde oxidase 4 (AAO4), is has been described that the role of the enzyme resides in the detoxification of aldehydes [25]. By plants, toxic aldehydes are generated under normal conditions, but their levels increase in response to stress. Since it has been demonstrated that the transcription levels of AAO4 were induced by hydrogen peroxide under stress conditions, it has been suggested that AAO4 plays a major role in the detoxification of aldehydes during increased hydrogen peroxide stress [25]. In humans, however, it remains speculative whether the enzyme plays a similar role and that its expression is also induced by increased levels of hydrogen peroxide. This needs to be investigated in future studies.

Unlike XOR, AOXs cannot use NAD^+^ as the final electron acceptor and they transfer the electrons generated during the enzymatic reaction only to molecular oxygen [26]. Consistent with this, the amino acid composition of the Q_423_ASRREDDIAK_433_ flexible loop I is very divergent in human AOX1 and XORs. In the crystal structure of human AOX1, the flexible loop II in proximity of the FAD pocket (T_1230_RGPDQ_1235_) is flipped by almost 180° to the relative in bXOR. Interestingly, the conformation of this loop is unique in human AOX1, and occupies almost the same position as the nicotinamide ring in the bXDH-NADH complex thereby blocking access to the isoaloxazine ring [11]. Due to this unique loop II orientation, we further investigated the NADH oxidase activity of hAOX1 wild-type. NADH is found in all living cells and it is the major source of the electrons transported by the mitochondria and responsible for the synthesis of ATP. Hepatic AOX preparations have been shown to oxidize NADH generating the reducing equivalents necessary for O_2_^•−^ production from molecular oxygen [19,27,28]. Our results, however, show that hAOX1 does not react with NADH. Hence, it is not possible that hAOX1 acts as a NADH oxidase which is capable of producing significant amounts of O_2_^•−^ in tissues. In previous studies mainly rat, rabbit and mouse enzymes were considered, for which a NADH oxidase activity has been obtained. Thus, the investigations on AOX enzymes need to be clearly divided into studies on the human enzyme, containing only one AOX orthologue and studies on the rodent enzymes, containing four orthologues with a significantly different substrate spectrum.

Our overall data provide new insights regarding the specific rate of oxygen radicals produced from the purified human AOX1 and in particular underline the crucial role of the residue Leu438 during the reaction FAD-oxygen implying a consequent high superoxide anion overproduction.

## Materials and methods

### Introducing SNP-based base pair exchanges into the hAOX1 gene by site directed mutagenesis

Site-directed mutagenesis of the hAOX1 variants was performed using the expression vector pTHcohAOX1 [13] as a template and base pair exchanges were introduced using the site directed mutagenesis kit (Quick Change, Agilent Technologies).

### Expression and purification of hAOX1 variants

The hAOX1 wild-type protein and generated variants were expressed and purified as described previously, with minor modifications [13]. The constructs pTHcohAOX1 (hAOX1 wild-type), pAF4 (G46E), pAF5 (G50D), pAF6 (G346R), pAF7 (H363Q), pAF8 (R433P), pAF9 (A437V), pAF10 (L438V), pAF11 (A439E), pAF12 (R1231H), pAF13 (K1237N) were transformed into *E*. *coli* TP1000 (Δ*mobAB*) cells [29]. For protein expression, *E*. *coli* cell cultures were grown at 30°C in LB medium supplemented with 150 μg/ml ampicillin, 1 mM sodium molybdate and 20 μM IPTG. Cells were harvested by centrifugation after 24 h of cell growth and resuspended in 50 mM sodium phosphate buffer, pH 8.0, containing 300 mM NaCl. After cell lysis the crude extract containing hAOX1 was first purified using a nickel-nitrilotriacetic acid resin (QIAGEN GmbH, Hilden, Germany) and then a size exclusion chromatography step using a Superdex 200 10/300 GL Column (GE Healthcare) as described previously [13].

### Metal quantification

The metal content of hAOX1 wild-type and variants was quantified by Inductively Coupled Plasma Optical Emission Spectroscopy (ICP-OES) on a Optima 2100 DV system (PerkinElmer Life and Analytical Sciences, Waltham, MA). 500 μL of purified hAOX1 (10 μM) and an equal volume of 65% nitric acid were mixed to wet ash the protein over night at 100°C overnight. The samples were diluted with 4 ml of water. A standard solution was used for calibration and quantification of the detected metals (Standard Solution XVI, Merck). The resulting mass concentrations were calculated and related as percent of protein saturated with molybdenum and iron corresponding to the two [2Fe2S] clusters.

### Steady-state kinetics

Steady state enzyme kinetics were performed with purified hAOX1 in 50 mM Tris buffer, pH 8.0, containing 200 mM NaCl and 1 mM EDTA at 25°C in a final volume of 500 μL. The substrate phenanthridine was used in a range of 1–100 μM and molecular oxygen was used as electron acceptor (in air-saturated buffer). The product phenanthridone was detected by absorbance at 321 nm. Total enzyme concentration varied between 100–200 nM, depending on the variant. Reactions were monitored over a range of 30 seconds. Activities were calculated using the molar extinction coefficients of 16,100 M^-1^cm^-1^ (at 600 nm) for 2,6-dichlorophenolindophenol (DCPIP), 1080 M^-1^cm^-1^ (at 420 nm) for potassium ferricyanide, or 6,380 M^-1^cm^-1^ (at 321 nm) for phenanthridinone. Bovine xanthine oxidase (bXO) activity was measured using a range of 0–100 μM xanthine solved in 0.1 M NaOH as substrate. Mean values with standard deviation were obtained from at least 3 independent measurements. k_cat_ values were normalized to 100% molybdenum content. The kinetic constants were obtained using the Michaelis-Menten equation by non-linear regression with the software Origin Pro 8.1G (Waltham, MA). The enzyme kinetics assays were performed on a Shimadzu UV-2401PC photometer.

### Superoxide production

The amount of superoxide produced during the enzymatic reactions was measured by monitoring the reduction of cytochrome c (Sigma-Aldrich) at 550 nm and 30°C. Reaction mixtures (500 μl) contained 200 μM cytochrome c and 0–100 μM phenanthridine in buffer Tris 50 mM, NaCl 200 mM and EDTA 1 mM (pH 8.0). The enzyme concentrations in the assay ranged from 100 to 200 nM depending on the variant used. Enzymes were added to the mixture to initiate the reaction and the absorbance change per min^-1^ was recorded with a spectrophotometer (Shimadzu UV-2600). Superoxide formation was calculated using an extinction coefficient of 21000 M^-1^cm^-1^ for cytochrome c [30] [31]. The extinction coefficients used for phenanthridone was 6,380 M^-1^cm^-1^. Superoxide dismutase (10 μg/ml), raloxifene (10 μM), or catalase (100 U/ml) were added as indicated.

### Quantification of the FAD cofactor

One milliliter of hAOX1 WT and variants (10 μM) was incubated with 150 μM of trichloroacetic acid (50% w/v) for 10 min on ice. The denatured protein was centrifuged at 14 000 g for 20 min, and the pellet was resuspended in 200 μl of trichloroacetic acid (5% w/v) and centrifuged again for 10 min. Both supernatants were combined, and 300 μl of 2 M K_2_HPO_4_ was added. The released FAD was detected photometrically on a Shimadzu UV-2401 PC photometer (Shimadzu Europa, Duisburg, Germany) at 450 nm. The FAD saturation in hAOX1 wild-type and variants was calculated from the specific extinction coefficient of free FAD (11,300 M^–1^ cm^–1^ at 450 nm) and from a calibration curve obtained from different concentrations of free FAD in solution.

### NADH reduction assay

hAOX1 wild-type and bXO were incubated with 100 μM of NADH under anaerobic conditions. UV–Vis spectra of hAOX1 and bXO were recorded over time (1, 10 and 20 min) in a range of 250–800 nm on a spectrophotometer (Shimadzu Europa, Duisburg, Germany) in 50 mM Tris, 200 mM NaCl and 1 mM EDTA buffer (pH 8.0). Total reduction of the protein sample was achieved by the addition of 1 mM of sodium dithionite.
